# Commentary: Are Children Like Werewolves? Full Moon and Its Association with Sleep and Activity Behaviors in an International Sample of Children

**DOI:** 10.3389/fped.2016.00094

**Published:** 2016-08-31

**Authors:** James S. Welsh

**Affiliations:** ^1^Department of Radiation Oncology, Stritch School of Medicine, Loyola University Chicago, Maywood, IL, USA

**Keywords:** full moon, lunacy, human behavior, pediatric psychology, perigee

The article, “Are Children Like Werewolves? Full Moon and Its Association with Sleep and Activity Behaviors in an International Sample of Children” by Chaput et al. (1) was quite fascinating and thought-provoking. However, there is a potential flaw in their research – and perhaps in all the medical literature on this subject.

Curiously, the study found a small but measurable difference in sleep duration in children as a function of the lunar cycle. Specifically, they found that nocturnal sleep duration is ~5 min shorter around full Moon compared to new Moon in their sample of children selected from around the world. One might reasonably speculate that this could be due to a brighter night sky during the full moon. To further evaluate this possibility, I would agree with the authors when they say, “Future experimental work is needed to determine if the human biology is in any way synchronized with the lunar cycle.” And that “Future research should also examine if the full moon may have a larger influence on subgroups of vulnerable children, e.g., those with mental disorders or physical ailments.”

However, I believe that it is important that any future research stratify the full Moons by relative proximity to Earth.

Our Moon does not orbit Earth in a perfect circle but rather in an ellipse. Thus, during every lunar orbit, there is a point in the ellipse when the Moon is closest to Earth (perigee) and a point when it is farthest from Earth (apogee). On average, the Moon is about 238,800 miles (384,300 km) from Earth. However, due to the elliptical shape of the lunar orbit, the absolute distance varies between 225,804 miles (363,396 km) at perigee and 251,968 miles (405,503 km) at apogee. Our Moon cycles through perigee and apogee approximately once each month but these points do not necessarily correlate with the lunar phases (new Moon, half Moon, full Moon, etc.). There are times, however, when the two cycles *are* synchronized and a full Moon would coincide with perigee or apogee.

The Moon’s gravitational pull on earth is strongest during perigee. The gravitational force exerted by the Moon on Earth, as calculated by Newton’s Law, is quite small. Nevertheless, the combined effects of the Moon and Sun do have a noticeable influence on our tides and twice per month we have alignment of the Sun, Earth, and Moon (i.e., a syzygy) giving us “spring tides” during new and full Moons. A few times per year when a full or new Moon coincides with lunar perigee, there are “perigean spring tides” thanks to this maximal gravitational impact of the Sun and Moon. The variation between maximum and minimum in gravitational attraction can be over 20%, resulting in slightly higher tides overall and greater variation in the high tide versus low tide. The absolute tidal change during a perigean spring tide is not really very much however, typically a few inches above an ordinary spring tide.

Perhaps more relevant to the paper by Chaput et al. (1) is that a full Moon during perigee (technically called a perigee-syzygy full Moon or colloquially a “supermoon”) can appear 12–4% larger and up to 30% brighter than a full Moon that is at its furthest from Earth (an apogee-syzygy full Moon or “micro moon”). Given this relatively large difference in apparent brightness, one could logically anticipate that the change in duration of sleep in children during a full moon observed by Chaput et al. could be amplified during super moons and minimized during micro moons.

Therefore, whenever any medical or behavioral research into the possible effects of lunar phase (such as a full Moon) is undertaken, whether the full Moon(s) under study are at lunar perigee or apogee or somewhere in between should be carefully recorded. Such subtleties have not consistently been reported in medical and behavioral studies of the influence of the full Moon. This could account for some of the inconsistencies in the reported results and the variable conclusions. For example, Cajochen et al. (2) reported that in 33 adults measured under controlled laboratory conditions, sleep duration diminished by about 20 min and sleep quality was decreased around the full Moon. These investigators did take nighttime brightness into account. Their results were consistent with those of Smith et al. (3) who also found a reduction of 25 min in sleep duration around full Moon in 47 adults similarly tested under controlled laboratory conditions. In contrast, in a large, Swiss, non-laboratory based study of adults Haba-Rubio et al. (4) demonstrated no significant impact of the lunar cycle on sleep. In children, Chaput et al. (1) in a 12-country study of children from around the world, recently, found that children slept about 5 min (1%) less around full Moon compared to new Moon. However, conflicting results were obtained by Sjödin et al. (5) when they observed that children slept slightly, but significantly, *longer* on average around full Moon and were somewhat less physically active during the day.

It is not clear that Chaput et al. (1) or any of the researchers took the relatively easily overlooked variable of apogee and perigee into their study of the influence of the full Moon, and perhaps this missing variable accounts for some of the discrepancies in the results. Whether the effect of a full Moon on sleep and other human behaviors is merely due to the apparent luminosity or another mechanism is unclear. The reproductive cycle of the Pacific Palolo worm [Palolo siciliensis (*Psychotria viridis* or *Eunice viridis*)], for instance, is chronologically tied in somehow with an annual cycle and lunar phases, but the exact mechanism remains uncertain. Specifically, spawning occurs at about 2:00 a.m. local time around the seventh night after the full Moon that follows the autumnal equinox in October with a second spawning occasionally on the neap tide of the last quarter Moon in November (6). It appears that lunar luminosity (i.e., brightness) is a key element in the reproductive cycle of this and similar marine organisms (7). Given that Chaput et al. (1) focused on sleep duration, and given that a full Moon during perigee can be nearly a third brighter than a full Moon at apogee, it certainly seems reasonable that this night-sky brightness could be playing a substantial role. As Chaput et al. (1) mentioned, further work on the potential influence of the lunar cycle on human behavior is warranted – but such future work should account for apogees and perigees.

It may very well be that when it comes to influence on human (and animal) behavior, not all full Moons are created equal!

## Author Contributions

The author confirms being the sole contributor of this work and approved it for publication.

## Conflict of Interest Statement

The author declares that the research was conducted in the absence of any commercial or financial relationships that could be construed as a potential conflict of interest.
